# Altered intrinsic thalamic network based on electroencephalography source-level analysis in poststroke epilepsy

**DOI:** 10.1097/MD.0000000000041886

**Published:** 2025-03-21

**Authors:** Dong Ah Lee, Junghae Ko, Bong Soo Park, Kang Min Park

**Affiliations:** a Department of Neurology, Haeundae Paik Hospital, Inje University College of Medicine, Busan, Republic of Korea; b Department of Internal medicine, Haeundae Paik Hospital, Inje University College of Medicine, Busan, Republic of Korea.

**Keywords:** electroencephalography, epilepsy, stroke, thalamus

## Abstract

This study aimed to investigate the alterations in the intrinsic thalamic network in patients with poststroke epilepsy (PSE) based on electroencephalography (EEG) source-level analysis. This retrospective observational study followed the STROBE guidelines. Thirty-nine patients with stroke and PSE and 34 patients with stroke without PSE were enrolled. These patients underwent EEG in a resting state. Source localization based on scalp electrical potentials was computed using the minimum norm imaging method and the standardized low-resolution brain electromagnetic tomography approach. To construct a functional connectivity matrix, the Talairach atlas was used to define the nodes belonging to the thalamus, and the coherence method was applied to measure brain synchronization as edges. The intrinsic thalamic network was analyzed using graph theory and compared between patients with and without PSE. EEG source-level analysis revealed notable differences in the intrinsic thalamic network between patients with and without PSE. From the undirected weighted connectivity matrix, the measure of modularity was lower in patients with PSE than in those without PSE (0.038 vs 0.106, *P* = .024). Additionally, modularity measures showed significant differences between the groups, as demonstrated by graph theoretical analysis using binary undirected graphs with a fixed density range of connections. This study is the first to demonstrate the alterations in the intrinsic thalamic network in patients with stroke with PSE compared to those without PSE based on EEG source-level analysis. These intrinsic thalamic network changes may be related to PSE development.

## 1. Introduction

In the development of epilepsy, cerebrovascular diseases account for approximately 11% of etiology, of which 70% to 85% are of ischemic origin.^[1]^ Conversely, the incidence rate of epilepsy after cerebrovascular disease is 3% to 30%, depending on the type of cerebrovascular disease.^[1]^ The prevalence of poststroke epilepsy (PSE) varies depending on the definition of early or late seizures or the subtypes of stroke.^[1–3]^ In general, seizures that occur within 1 week of stroke onset are defined as early poststroke seizures, and those that occur later are classified as late poststroke seizures.^[4]^ According to the International League Against Epilepsy proposal, late poststroke seizure can be diagnosed as epilepsy by satisfying “enduring alteration in the brain that increases the likelihood of future seizures.”^[5]^

Previous studies have focused on clinical factors that predict PSE development after stroke onset. The possibility of PSE development can increase in the presence of hemorrhagic stroke, high severity of clinical symptoms, cortical involvement, young age onset, and early symptomatic seizures.^[3,6–8]^ However, these clinical factors alone cannot clearly predict PSE. After stroke occurs, structural changes in the brain and brain network and metabolism changes occur, and these changes are also associated with PSE development.^[1]^ It is also related to other chronic factors, such as patients’ comorbidities and current medications; temporary factors, including early poststroke seizure and peripheral infection; and polymorphism of *CD40* and aldehyde dehydrogenase 2.^[1,9]^

Epilepsy is a disorder of network dysfunction. The methodological frameworks of functional brain connectivity and graph theory have been widely used to evaluate interictal brain network abnormalities in patients with epilepsy.^[10]^ Of the modalities investigating functional brain connectivity, electroencephalography (EEG) has the advantage of identifying functional brain network in “resting state.”^[11]^ The “resting state” is considered to reflect the intrinsic activity of the brain, and the process of information in resting state is encoded on significantly short time scale from milliseconds to seconds. Thus, EEG is a remarkably suitable tool for functional network analysis because it has excellent time resolution.^[12]^ Recent studies have revealed that using EEG-based graph neural networks to classify first-episode schizophrenia, chronic schizophrenia, and healthy controls (HC) outperforms the use of support vector machines.^[13]^ Furthermore, recent studies have revealed that an EEG-based multi-band joint graph convolution network can be used for the individual-level prediction of anxiety and depression disorders, outperforming traditional clinical scale assessments by integrating brain functional connectivity across multiple frequency bands.^[14]^ EEG has been used in the field of stroke studies to predict functional outcome, mortality, and cognitive decline after stroke.^[15]^ However, no studies have investigated the alterations of functional networks in patients with PSE based on EEG.

The circuitry of the focal seizure consists of at least 3 states, including seizure initiation, buildup, and spread.^[16]^ This circuitry of seizure is involved in complex brain network, and subcortical structures, including the thalamus, caudate nucleus, or other basal ganglia, are key components of this brain network. In particular, the thalamus plays a key role in the transition from interictal to ictal and in the stage of spreading and recruitment of seizures.^[17]^ Therefore, we can assume that the thalamus also plays a significantly important role in PSE. To elucidate the role of the thalamus in the epilepsy network, previous studies have mainly used functional magnetic resonance imaging, diffusion tensor imaging, or stereo EEG because the thalamus is 1 of the subcortical structures.^[17–19]^ Scalp EEG, which is mainly used in the clinical practice field, can detect the sensor-level signal and does not directly reflect the signal from the thalamus. Thus, source reconstruction methods are required to study the signal of the thalamus using a scalp-recorded EEG.^[12]^

This study aimed to assess alterations in the intrinsic thalamic network using source-level EEG analysis by comparing patients with stroke and PSE to those without PSE. We hypothesized that there would be significant differences in the intrinsic thalamic network between patients with and without PSE.

## 2. Methods

### 2.1. Participants

This study received approval from the Institutional Review Board of Haeundae Paik Hospital. All participants provided written informed consent before enrollment. A total of 39 patients with stroke and PSE and 34 patients with stroke without PSE were enrolled in this study. PSE was defined as the occurrence of at least 1 unprovoked seizure in the late poststroke period. Late poststroke seizures were defined as those occurring within the timeframe of 1 week to 2 years after stroke onset.^[8,20]^ All patients were newly diagnosed with PSE at our institution and had no history of seizures before their stroke. Patients in the non-PSE group had no prior diagnosis of epilepsy or seizures before stroke onset. Additionally, none of the patients in either group had a history of psychiatric disorders, developmental conditions, or other severe debilitating diseases.

Clinical data were collected for all participants, including sex and age at the time of EEG, and stroke etiology based on the Trial of ORG 10172 in Acute Stroke Treatment classification (large-artery atherosclerosis, cardioembolism, small-artery occlusion, other causes, or undetermined origin).^[21]^ Other recorded variables included the affected stroke hemisphere and location, initial National Institutes of Health Stroke Scale (NIHSS) score,^[22,23]^ presence of hemorrhagic transformation, comorbid conditions (atrial fibrillation, hypertension, diabetes mellitus, dyslipidemia, or others), time interval between stroke onset and EEG acquisition, and occurrence of seizures.

### 2.2. Electroencephalography acquisition

EEG recordings were obtained from all stroke patients while they were awake and in a resting state with their eyes closed. The recordings were obtained using a standardized EEG system (TWin® EEG software system) with consistent methodologies across all participants. Trained technical staff performed the EEG acquisition using gold electrodes applied with electrode paste. A total of 23 electrodes (Fp1, Fp2, F7, F8, T1, T2, T3, T4, T5, T6, O1, O2, F3, F4, C3, C4, P3, P4, Cz, Pz, Oz, A1, and A2) were positioned in accordance with the international 10 to 20 system. Electrode impedance was maintained below 5 kΩ throughout the recordings. The EEG signals were sampled at a frequency of 250 Hz, and each recording session lasted a minimum of 30 minutes. EEG acquisition followed the same methodology as described in our previous article.^[24]^

### 2.3. Electroencephalography preprocessing and source modeling

The analysis of EEG was conducted using Curry software (version 8). During data processing, EEG signals were referenced to an average. A band-pass filter was applied, with a low cutoff at 1.0 Hz and a high cutoff at 30.0 Hz. The EEG recordings were manually examined, and 3-second epochs were selected, ensuring the presence of alpha activity with maximal voltage in posterior regions while excluding artifacts or epileptiform discharges. The selection of epochs was carried out by DA Lee. Sources were subsequently computed based on their scalp electrical potentials using a minimum norm imaging method, which estimated the amplitude of brain sources distributed across the brain, and the standardized LOw-REsolution brain Electromagnetic TomogrAphy (sLORETA) approach. To construct a functional connectivity matrix, the Talairach atlas was utilized to define nodes, while the coherence method was applied to assess brain synchronization, represented as edges. Among various nodes, only 14 nodes corresponding to the thalamus were selected to analyze the intrinsic thalamic network (Supplementary File 1, Supplemental Digital Content, http://links.lww.com/MD/O581).

### 2.4. Graph theoretical analysis

Graph theoretical analysis was conducted using the BRAPH software.^[25]^ Functional connectivity metrics were computed from the undirected weighted connectivity matrix, including average degree, average strength, radius, diameter, characteristic path length, global efficiency, local efficiency, mean clustering coefficient, transitivity, modularity, and the small-worldness index.^[26]^ These measures were analyzed and compared between patients with and without PSE. Additionally, binary undirected graphs were employed for further analysis at a fixed connection density (ranging from 15% to 95% in 5% increments) when statistically significant differences were identified in the weighted connectivity analysis.

### 2.5. Statistical analyses

The clinical characteristics of patients with and without PSE were analyzed using the chi-squared test or an independent Student *t* test, as appropriate. All statistical analyses were performed using MedCalc® Statistical Software (version 20.014, MedCalc Software Ltd., Ostend, Belgium; https://www.medcalc.org; 2021). To assess differences in functional connectivity, nonparametric permutation tests with 1000 permutations were performed. A *P*-value of <0.05 was considered statistically significant for all analyses. Categorical variables were reported as frequencies and percentages, while numerical variables were expressed as mean ± standard deviation.

## 3. Results

### 3.1. Patient demographics and clinical features

No significant differences were observed between patients with and without PSE in terms of age, sex, etiology of stroke, side and location of stroke, NIHSS score, presence of hemorrhagic transformation, or comorbidities. However, the time interval between stroke onset and EEG acquisition differed significantly, with a longer interval in patients with PSE compared to those without PSE (61.0 vs 5.0 months, *P* < .001) (Table 1).

**Table 1:** Differences of the clinical characteristics between patients with and without PSE.

	Patients with PSE (N = 39)	Patients without PSE (N = 34)	*P*-value
Age, yr (±SD)	74.1 (±10.8)	75.6 (±14.1)	.582
Male, N (%)	24 (61.5)	16 (47.1)	.218
Stroke etiology, N (%)			
Cardioembolism	17 (43.6)	15 (44.1)	.066
Large-artery atherosclerosis	3 (7.7)	6 (17.6)	
Small-vessel occlusion	0 (0)	1 (2.9)	
Undetermined etiology	12 (30.7)	12 (35.3)	
Other determined etiologies	7 (17.9)	0 (0)	
Stroke side, N (%)			
Right	18 (46.1)	12 (35.3)	.227
Left	15 (38.5)	11 (32.4)	
Bilateral	6 (15.4)	11 (32.4)	
Stroke location, N (%)			
Frontal lobe	21 (53.8)	19 (55.9)	.186
Temporal lobe	19 (48.7)	16 (47.1)	
Parietal lobe	20 (51.3)	14 (41.2)	
Occipital lobe	3 (7.7)	7 (20.6)	
Subcortex or brainstem	8 (20.5)	14 (41.2)	
Cerebellum	3 (7.7)	9 (26.5)	
NIHSS score (±SD)	9.5 (±5.6)	12.0 (±7.7)	.236
Hemorrhagic transformation, N (%)	9 (23.1)	10 (29.4)	.442
Comorbidity, N (%)			
Hypertension	21 (53.8)	23 (67.6)	.627
Diabetes	10 (25.6)	12 (35.3)	
Dyslipidemia	5 (12.8)	5 (14.7)	
Atrial fibrillation	12 (30.7)	13 (38.2)	
Others	6 (15.4)	15 (44.1)	
Time interval between stroke onset and EEG, months (±SD)	61.0 (±77.1)	5.0 (±7.3)	<.001
With focal to bilateral tonic-clonic seizures, N (%)	30 (76.9)		

EEG = electroencephalography, NIHSS = National Institute of Health Stroke Scale, PSE = poststroke epilepsy, SD = standard deviation.

### 3.2. Intrinsic thalamic network

**Figure 1.**
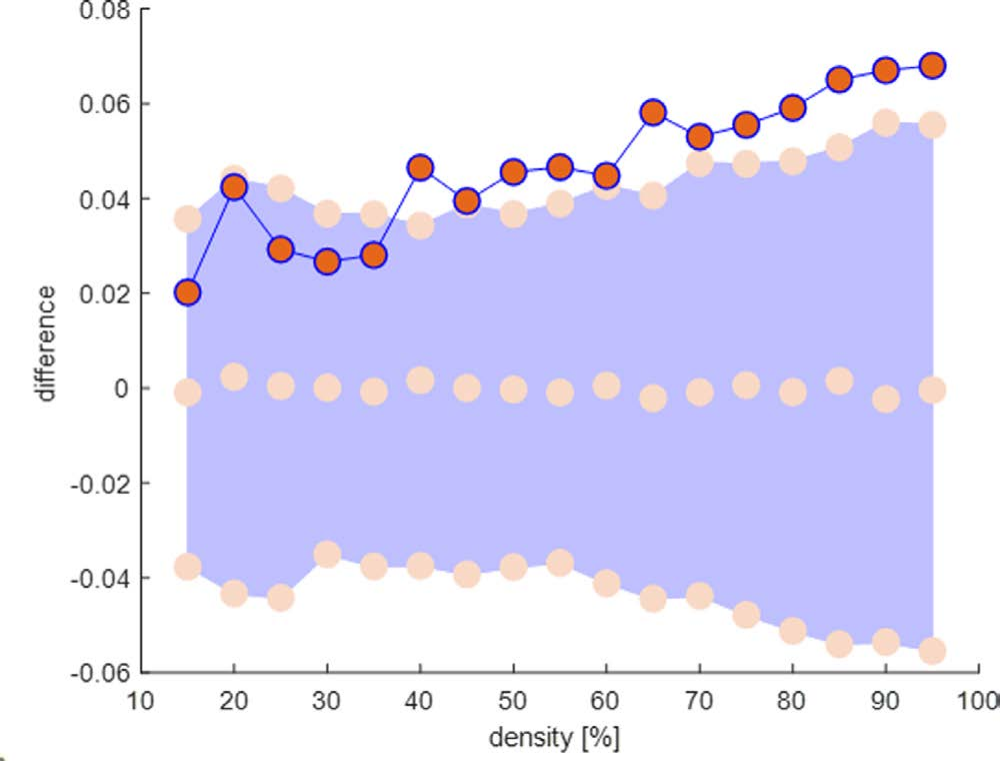
Differences in the functional connectivity measure of modularity between patients with and without poststroke epilepsy (PSE). The violet area represents the 95% confidence intervals of the between-group differences obtained for 1000 permutation tests at each density. Red circles indicate differences in the functional connectivity measure of modularity between patients with and without PSE. There are significant differences in the modularity between the groups.

**Figure 2.**
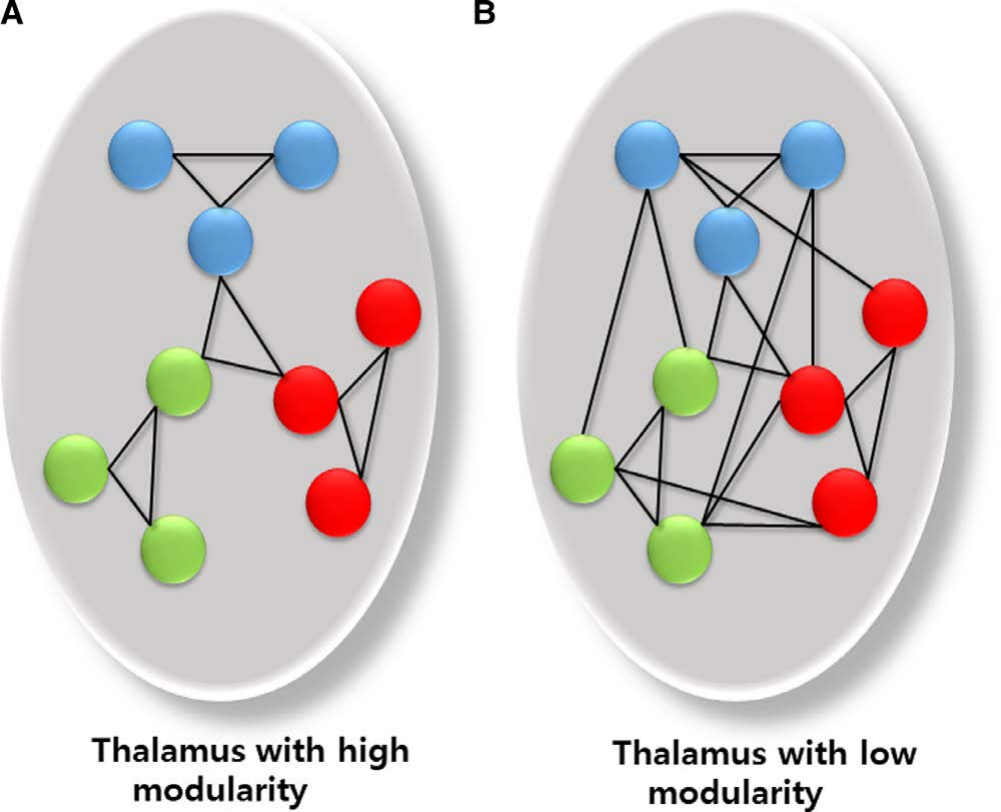
Schematic representation of intrinsic thalamic networks with high and low modularity. This figure represents a schematic illustration of the thalamus. The circular shapes denote nodes, while the connecting lines indicate edges. Panels (A and B) depict thalamic intrinsic networks with high modularity and low modularity, respectively.

Table 2 presents the differences in the intrinsic thalamic network between patients with and without PSE, as analyzed using EEG source-level data. Among the functional connectivity metrics, modularity was the only measure that significantly differed between the groups, with patients with PSE exhibiting lower modularity compared to those without PSE (0.038 vs 0.106, *P* = .024). Additionally, graph theoretical analysis using binary undirected graphs at fixed density connections confirmed significant differences in modularity between the groups (Figs. 1 and 2) (Supplementary File 2, Supplemental Digital Content, http://links.lww.com/MD/O582).

**Table 2:** Difference of intrinsic thalamic network based on EEG source-level analysis between patients with and without PSE.

	Patients with PSE (N = 39)	Patients without PSE (N = 34)	Difference	CI lower	CI upper	*P*-value
Average degree	11.861	11.130	–0.731	–0.901	0.983	.097
Average strength	11.700	10.989	–0.711	–1.016	0.978	.113
Radius	1.018	1.019	0.002	–0.008	0.008	.385
Diameter	1.066	1.068	0.003	–0.030	0.037	.487
Characteristic path length	1.014	1.014	–0.001	–0.006	0.005	.442
Global efficiency	0.900	0.845	–0.055	–0.074	0.075	.097
Local efficiency	3.309	3.208	–0.101	–0.166	0.173	.166
Mean clustering coefficient	0.976	0.983	0.008	–0.012	0.012	.167
Transitivity	1.479	1.481	0.001	–0.008	0.009	.398
Modularity	0.038	0.106	0.068	–0.058	0.058	.024*
Assortative coefficient	0.142	0.152	0.009	–0.182	0.170	.434
Small-worldness index	1.147	1.307	0.160	–0.169	0.172	.064

CI, 95% = confidence interval of the difference between the groups, EEG = electroencephalography, PSE = poststroke epilepsy.

* *P* < .05.

However, the other functional connectivity measures, including the average degree, average strength, radius, diameter, characteristic path length, global efficiency, local efficiency, mean clustering coefficient, transitivity, and small-worldness index, did not differ between the groups.

## 4. Discussion

The present results demonstrated that there were significant alterations in the intrinsic thalamic network based on EEG source-level analysis in patients with stroke with PSE compared to those without PSE. We found that modularity was lower in patients with stroke with PSE than in those without PSE. This suggests that the thalamus plays an important role in PSE development.

The thalamus is a relay center for brain signaling, and the connection between different thalamic nuclei and the neocortex has been well reported.^[27]^ The importance of the thalamus in the development of epilepsy has been demonstrated in a previous study. A study with a model of acquired absence epilepsy demonstrated that the alteration in GABA_A_ receptor properties in reticular nuclei and ventrobasal relay nuclei of the thalamus generated epilepsy.^[28]^ Furthermore, thalamic involvement in the epileptic network and epileptogenic cortical lesion form a network with the subcortical structure, which is also related to clinical outcome in epilepsy.^[17,29]^ In a previous study of patients with temporal lobe epilepsy, patients who had a broad epileptogenic network involving extra-temporal regions, especially the thalamus as a hub, had poor surgical outcome.^[29]^ It was consistent with another previous study suggesting that disrupted global network properties were related to large abnormal hubs, including the thalamus.^[30]^

Interestingly, when a brain lesions such as a stroke, occurs in the cortex, abnormalities also occur in the thalamus connected to it.^[31]^ An animal experimental model showed that in the acute stroke phase (within 1 week), the cell death of corticothalamic neurons of the injured cortex caused cell death of connected thalamocortical neurons. Ipsilateral thalamocortical neurons exhibited increased intrinsic membrane excitability and enhanced rhythmogenic properties 7 to 14 days after stroke. This hyperexcitable loop has the potential to generate epilepsy. Furthermore, selective optical inhibition of thalamocortical neurons effectively disrupts seizures. This study proves that the thalamus plays a critical role in seizure maintenance in acquired epilepsy and identifies that a secondarily damaged thalamus can be targeted to stop seizures. These studies suggest that the thalamus contributes to the development of focal epilepsy, consistent with our findings.

Graph theory is 1 of the most representative analysis methods for brain connectivity and network, which is a mathematical framework that defines a network as nodes and edges.^[32,33]^ One of the characterizing topological properties of a healthy human network is small-worldness network.^[26]^ The small-world network optimally balances wiring cost and information flow efficiency by ensuring that each pair of nodes is connected through the shortest possible path with a minimal number of edges.^[26]^ The small-world network achieves an optimal balance between segregation, which reflects the formation of specialized communities within the network, and integration, which represents the efficiency of global information transfer.^[34]^ Module presents groups of densely interconnected nodes.^[32]^ The modularity is 1 of the common parameters, which quantifies the properties of topological segregation and is used to sub-partition in brain network.^[35]^ In present study, we found the altered intrinsic thalamic modularity in patients with PSE. Previous studies also showed that functional network in patients with focal epilepsy had altered modularity compared to HC.^[36–38]^ The strong modular reorganization of hubs in patients with frontal lobe epilepsy was demonstrated, and abnormalities in the whole brain network could be originated from the abnormal modularity.^[38]^ Moreover, this abnormality of modularity was related to functional outcomes, such as cognitive impairment, aphasia, and motor deficit in poststroke conditions.^[39–41]^ Greater poststroke network fragmentation in the left hemisphere and higher modularity index were associated with more severe chronic aphasia.^[41]^ In this present study, changes in the intrinsic thalamic network are related to thalamic excitation, which is considered to be related to epilepsy development.

The advantage of EEG for the measurement of brain function is that it can reflect the mechanisms of communication among neural assemblies, such as coherence, synchronization and phase locking.^[42]^ However, the EEG has limited spatial resolution due to the small number of channels and the challenges posed by the inverse problem. Several solutions have been introduced to solve the inverse problem that occurs during the reconstruction of EEG from the sensor-level to the source-level analysis, although no unique solution exists for this problem.^[12]^ Moreover, source-level EEG analysis does not fully resolve issues related to field spread, where multiple electrodes detect signals from the same source, or volume conduction, which causes signal blurring due to the conductive properties of the human head. Thus, it can be helpful to perform connectivity analysis along with source-level analysis.^[12]^ In this study, we selected the combination of sLORETA for inverse solution and coherence to analyze source-level functional connectivity. Coherence measures the linear relationship between the amplitude of 2 signals in the frequency domain.^[43]^ It is a commonly used and relatively straightforward approach to obtain an impression about functional networks; the coherence of 2 nodes A and B means that the coupling of neuronal oscillation or synchronizing input is received from the third node.^[42]^

This study has several limitations. First, the limited sample size and single-center design may restrict the generalizability of the findings. Second, the cross-sectional design necessitates an investigation into whether longitudinal differences exist in the intrinsic thalamic network between patients with and without PSE. Third, there was a discrepancy in the time interval between stroke onset and EEG acquisition between the 2 groups. In patients with PSE, EEG was performed after experiencing more than 1 late-onset seizure, whereas in most patients without PSE, EEG was conducted within a few months after stroke. However, potential confounding effects of anti-seizure medications were ruled out, as all EEG recordings were obtained before medication initiation. Lastly, the network measures of the intrinsic thalamic network were analyzed at the group level, as individual-level data were not available. As a result, correlation analyses with clinical factors could not be performed.

To our knowledge, this is the first study to demonstrate alterations in the intrinsic thalamic network using EEG source-level analysis in patients with stroke and PSE compared to those without PSE. These changes in the intrinsic thalamic network may be associated with the development of PSE.

## Acknowledgments

This work was supported by the National Research Foundation of Korea (NRF) grant funded by the Korea government (MSIT) (No. RS-2023-00209722).

## Author contributions

**Conceptualization:** Kang Min Park.

**Data curation:** Dong Ah Lee, Junghae Ko, Bong Soo Park.

**Formal analysis:** Kang Min Park.

**Methodology:** Kang Min Park.

**Project administration:** Kang Min Park.

**Software:** Dong Ah Lee, Kang Min Park.

**Supervision:** Kang Min Park.

**Validation:** Dong Ah Lee, Kang Min Park.

**Visualization:** Junghae Ko, Bong Soo Park.

**Writing – original draft:** Dong Ah Lee, Junghae Ko, Bong Soo Park, Kang Min Park.

**Writing – review & editing:** Kang Min Park.

## Supplementary Material

SUPPLEMENTARY MATERIAL
