# Supplementary material for: Altered intrinsic thalamic network based on electroencephalography source-level analysis in poststroke epilepsy
Source: Medicine (Baltimore). 2025 Mar 21;104(12):e41886. doi: 10.1097/MD.0000000000041886 (PMC11936616; doi:10.1097/MD.0000000000041886)
Supplement: SUPPLEMENTARY MATERIAL [file medi-104-e41886-s001.docx]

**Supplementary 1.** The nodes belonging to the thalamus are selected from among several nodes

| X coordinate | Y coordinate | Z coordinate | Side | Thalamic nuclei |
| --- | --- | --- | --- | --- |
| -6.33 | -9.40 | 13.85 | Left Cerebrum | Anterior Nucleus |
| -12.75 | -18.42 | 19.77 | Left Cerebrum | Lateral Dorsal Nucleus |
| -5.57 | -15.33 | 13.67 | Left Cerebrum | Medial Dorsal Nucleus |
| -7.37 | -17.84 | 17.45 | Left Cerebrum | Midline Nucleus |
| -1.70 | -31.55 | 13.03 | Left Cerebrum | Pulvinar Nucleus |
| -14.26 | -19.45 | -1.42 | Left Cerebrum | Ventral Posterior Medial Nucleus |
| 9.44 | -14.99 | 19.70 | Right Cerebrum | Anterior Nucleus |
| 11.81 | -19.44 | 19.34 | Right Cerebrum | Lateral Dorsal Nucleus |
| 14.45 | -21.02 | 19.96 | Right Cerebrum | Lateral Posterior Nucleus |
| 6.71 | -20.54 | 3.71 | Right Cerebrum | Medial Dorsal Nucleus |
| 9.07 | -17.99 | 18.97 | Right Cerebrum | Midline Nucleus |
| 24.65 | -33.89 | 6.78 | Right Cerebrum | Pulvinar Nucleus |
| 18.23 | -20.38 | -2.66 | Right Cerebrum | Ventral Posterior Lateral Nucleus |
| 16.15 | -19.11 | -1.80 | Right Cerebrum | Ventral Posterior Medial Nucleus |
