# Supplementary material for: Altered intrinsic thalamic network based on electroencephalography source-level analysis in poststroke epilepsy
Source: Medicine (Baltimore). 2025 Mar 21;104(12):e41886. doi: 10.1097/MD.0000000000041886 (PMC11936616; doi:10.1097/MD.0000000000041886)
Supplement: SUPPLEMENTARY MATERIAL [file medi-104-e41886-s002.docx]

**Supplementary 2.** Differences in the measure of modularity between patients with and without post-stroke epilepsy

| **Density** | **Patients with PSE (N=39)** | **Patients without PSE (N=34)** | **Difference** | **CI lower** | **CI upper** | ***p*-value** |
| --- | --- | --- | --- | --- | --- | --- |
| 15 | 0.533 | 0.554 | 0.020 | -0.038 | 0.036 | 0.168 |
| 20 | 0.471 | 0.513 | 0.042 | -0.043 | 0.044 | 0.059 |
| 25 | 0.402 | 0.431 | 0.029 | -0.044 | 0.042 | 0.145 |
| 30 | 0.356 | 0.382 | 0.027 | -0.035 | 0.037 | 0.125 |
| 35 | 0.307 | 0.335 | 0.028 | -0.038 | 0.037 | 0.108 |
| 40 | 0.278 | 0.324 | 0.047 | -0.038 | 0.034 | 0.011 |
| 45 | 0.256 | 0.295 | 0.040 | -0.039 | 0.039 | 0.047 |
| 50 | 0.220 | 0.265 | 0.046 | -0.038 | 0.037 | 0.025 |
| 55 | 0.190 | 0.237 | 0.047 | -0.037 | 0.039 | 0.025 |
| 60 | 0.174 | 0.218 | 0.045 | -0.041 | 0.043 | 0.041 |
| 65 | 0.142 | 0.200 | 0.058 | -0.044 | 0.041 | 0.009 |
| 70 | 0.127 | 0.180 | 0.053 | -0.044 | 0.048 | 0.026 |
| 75 | 0.106 | 0.162 | 0.056 | -0.048 | 0.047 | 0.027 |
| 80 | 0.087 | 0.146 | 0.059 | -0.051 | 0.048 | 0.017 |
| 85 | 0.067 | 0.132 | 0.065 | -0.054 | 0.051 | 0.022 |
| 90 | 0.049 | 0.116 | 0.067 | -0.054 | 0.056 | 0.024 |
| 95 | 0.038 | 0.106 | 0.068 | -0.055 | 0.056 | 0.024 |

PSE: post-stroke epilepsy, CI: 95% confidence interval of the difference between the groups
